# The State Board of Medical Examiners

**Published:** 1901-08

**Authors:** 


### TEXAS MEDICAL JOURNAL.

AUSTIN, TEXAS.

A MONTHLY JOURNAL OF MEDICINE AND SURGERY.

EDITED AND PUBLISHED BY
F. E. DANIEL, M. D.

ASSOCIATE EDITOR:
WITTEN BOOTH RUSS, M. D.,
San Antonio, Texas.

Published Monthly at Austin, Texas. Subscription price $1.00 a year in advance.

Eastern Representative: John Guy Monihan, St. Paul Building, 220 Broadway, New York City.

Official organ of the State Association of Health Officers, the West Texas Medical Association, the Houston District Medical Association, the Austin District Medical Society, the Brazos Valley Medical Association, the Galveston County Medical Society, and several others.

### THE STATE BOARD OF MEDICAL EXAMINERS.

For the first time in the history of Texas the State has a board of medical examiners for licensing physicians, surgeons and professional midwives. The board met and organized July 23, as elsewhere stated, and will hold the first business meeting October 1 (first Tuesday), at Austin.

These gentlemen have before them a great and responsible task. That they will conscientiously meet it, and bring to its discharge professonal ability, zeal and earnestness, with an eye single to the best interests of the public and the profession, is not to be questioned. The standard of requirements will be high, and merit and professional and private character alone will be considered. No more "licenses granted while the train waits." It is the duty of every physician, a duty to the State, to the Board, to himself, his family and the public, to co-operate with the Board and the law officers to see that no one is permitted to practice medicine in violation of the law. Physicians should not, as is too frequently the case, refrain from bringing to the attention of the county or district attorney every case where a person not exempt from this act or licensed by the Board is practicing medicine, through fear of being accused of envy, jealousy, or the desire to get rid of a competitor; and the Board calls upon the profession, and the Red-Back echoes the call and impresses it upon its readers, to be active in the discharge of this duty, unpleasant though it be. The Board should, and will doubtless enlist the cordial co-operation of the Attorney-General, who should call upon all county and district attorneys to promptly present all offenders to the grand jury. A license from this Board bears the seal of the State, and will be the requisite to the privilege of practicing in Texas.

The Board have already issued instructions, in circular form, setting forth how an applicant for license should proceed. They have mailed five thousand copies of the following letter to Texas physicians, as per Polk's Directory:

THE BOARD OF MEDICAL EXAMINERS FOR THE STATE OF TEXAS.

MEMBERS OF BOARD.

J. T. WILSON, SHERMAN, PRESIDENT.
S. R. BURROUGHS, BUFFALO, V. PRESIDENT.

| | |
|---|---|
| J. C. JONES, GONZALES. | J. W. SCOTT, HOUSTON. |
| F. PASCHAL, SAN ANTONIO. | J. H. EVANS, PALESTINE. |
| J. H. REUSS, CUERO. | D. J. JENKINS, DAINGERFIELD. |

M. M. SMITH. AUSTIN, SECRETARY AND TREASURER.

OFFICE OF THE SECRETARY, AUSTIN, TEXAS, July 25, 1901.

*Doctor* ........................,
........................*Texas.*

DEAR DOCTOR:—The Board of Medical Examiners for the State of Texas requires all physicians in the State who are practicing medicine upon the registration of their diplomas since the year 1891, to furnish the secretary of said board with a certificate from the dean of the college from which the applicant graduated and the year of graduation, etc.; also it is necessary for the district clerk from the county where applicant is practicing to certify that said diploma was registered in his office in due form.

When this data has been received by the secretary, the board will take up the question and if the data is satisfactory, then a certificate will be issued without cost to the applicant, and upon registering the same in the district clerk's office and the payment of fifty cents, the applicant will have complied with the new law and can practice medicine and surgery legally in the State of Texas.

You are advised to give this matter your prompt attention and thereby conform to the requirements of the law.

Please find enclosed blank forms which you will have filled out by the district clerk and the dean of the college where you graduated. It will not be necessary for you to submit your diploma to the board.

M. M. Smith, Secretary.

In addition to the above they have sent out, or will send on request, (1) a blank form of application for license based on license from other State Boards; (2) Application for examination by Texas State Board; (3) Certificate of moral character; (4) Rules adopted by the Board for conducting examinations, etc., etc. We regret that we have not room to reproduce these documents. They may be had upon application to the Secretary at Austin.

